# An Investigation on the Acetone and Ethanol Vapor-Sensing Behavior of Sol–Gel Electrospun ZnO Nanofibers Using an Indigenous Setup

**DOI:** 10.1021/acsomega.3c06744

**Published:** 2023-12-11

**Authors:** Niranjan N Prabhu, Basavannadevaru Shivamurthy, Srinivasan Anandhan, Bharathipura Venkataramana Rajendra, Jagadeesh Chandra Regati Basanna, Manu Srivathsa

**Affiliations:** † Department of Mechanical & Industrial Engineering, Manipal Institute of Technology, Manipal Academy of Higher Education, Manipal 576104, India; ‡ Department of Metallurgical and Materials Engineering, National Institute of Technology-Karnataka, Srinivas Nagar, Mangalore 575025, India; § Department of Physics, Manipal Institute of Technology, Manipal Academy of Higher Education, Manipal 576104, Karnataka, India; ∥ Department of Electronics and Communication Engineering, Manipal Institute of Technology, Manipal Academy of Higher Education, Manipal 576104, Karnataka, India

## Abstract

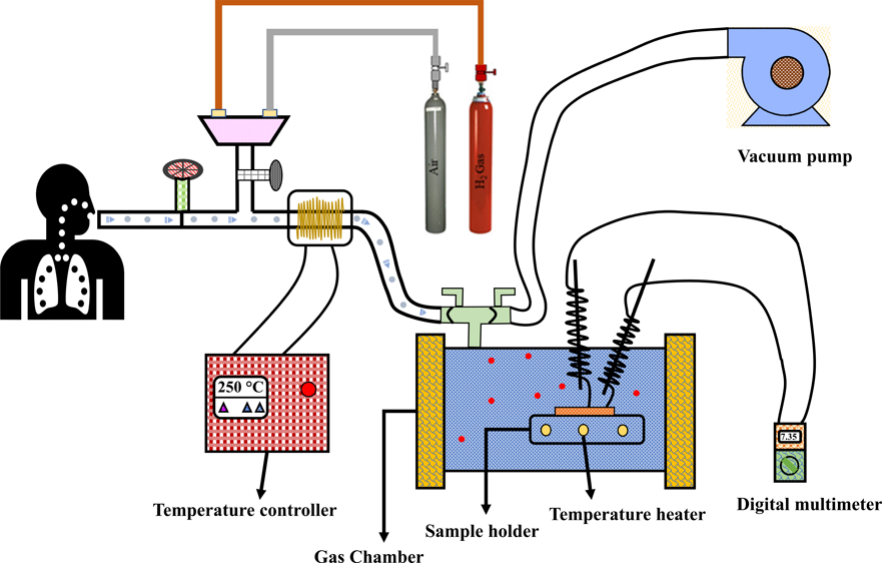

The calibration is essential for accuracy, repeatability, and continuous trouble-free operation of gas sensors with safety. Most gas sensors are fabricated using metal oxide nanomaterials in different structures such as films, coating, or nanofibers. Therefore, a device in the sensor manufacturing industry is necessary to test, calibrate, and optimize metal oxide structures. In this point of view, a simple device is developed to test and estimate the sensing response, response time, and recovery time of nanostructures. The sol–gel method was used to produce nanofibers through electrospinning. An average fiber diameter of 245 nm was obtained after pyrolysis at 600 °C. The structure and composition of ZnO nanofibers are confirmed by X-ray diffraction, scanning electron microscopy, and Brunauer–Emmett–Teller. The trials were taken using ZnO nanofibers in the presence of acetone and ethanol vapor, and the results were reported. High response (31.74), rapid response (40 s), and recovery (30 s) times have been achieved for ethanol gas to 50 ppm concentration test gas at an optimal temperature of 260 °C. The results obtained from the trials are compared with the literature results, which are in line with the values presented by the various researchers. Due to the low cost, easy maintenance, and accuracy, this device is recommended in metal oxide sensor development industries and laboratories.

## 1 Introduction

Metal oxides (MOs) have gained a lot of attention as sensing materials for detecting toxic gases and volatile organic compounds (VOCs).^1−3^ Particularly, MO nanofibers (NFs) become an attractive material because of their physical properties, such as compact size and high specific surface area. The directional strength and flexibility of NFs are an added advantage to the system in addition to its functional properties.^4^ Because of the design flexibility and high specific surface area, it has a good response, low cost, long-term stability, and increased sensitivity. Hence, NFs have high potential applications.^5−7^ Ceramic NFs with perovskite-type mixed MOs have attracted keen interest over the years owing to their unique physical and chemical properties.^8^ Zinc with titanium oxide, in particular has been widely researched for various applications such as catalytic sorbent for desulfurizing hot coal gases,^9^ microwave dielectric ceramics,^10^ and gas sensors^11^ for detecting ethanol, carbon monoxide, and nitric oxide. Yadawa et al.^12^ observed the change in morphology of ZnO thin films deposited on glass and quartz substrates after doping TiO_2_. The effect of their photocatalytic activity under visible light irradiation was investigated. The pyrolyzed quartz sample showed vertical crystalline morphology compared to wrinkled films on glass substrates. This was attributed to spreading less viscous liquid film against the more viscous film during the spin-coating process and to the surface energy difference between the ZnO and TiO_2_ crystallites. Also, more surface states were observed for quartz samples as compared with glass sample films under visible light illumination. This is due to the free crystallite surfaces, which introduce localized gap states that help absorb visible light, improving higher photocatalytic activity. Perween and Ranjan^13^ observed that sol–gel electrospinning is an efficient technique to produce zinc titanate (ZnTiO_3_) powders of nanoparticles followed by pyrolysis helped in improving the photocatalytic activity under visible light illumination. The XRD results confirmed the hexagonal structure of ZnTiO_3_. The BET surface area confirmed the nanoporous structure of the nanoparticles. The enhanced visible light photocatalytic activity is due to the enhanced surface area and larger carrier lifetimes of the nanopowder.

It is found that the abnormal concentration of gases in human breath indicates a certain possibility of diseases, which is reported in the clinical study literature.^14,15^ Hence, medical researchers proposed identifying a few diseases by analyzing the gas concentration in human breath. This technique is cheaper, faster, and more convenient than conventional tests. In this direction, few researchers reported using MO NFs based gas sensors to detect the concentration of various gases in human breath and diagnose the relevant disease. The permissible concentration limit of various gases in the human breath and abnormal various gas concentrations related to the predicted disease are reported in Table 1.

**Table 1:** Diseases Caused by Various Gases

gas in breath	permissible concentration (ppb)	probable diseases due to abnormal concentration	references
nitric oxide	24–26	asthma and cardiovascular sickness	(16)
hydrogen sulfide	7–16	asthma and airway inflammation	(17)
acetone	0.8–1	diabetes	(18)
isoprene	105	lung cancer	(19)
ammonia	250–255	kidney failure, peptic ulcer	(20)

From the above discussion, in this work, we made an attempt to develop a device to investigate the gas-sensing performance of zinc oxide (ZnO) NFs in the sensor manufacturing industry. The ZnO NFs were synthesized; their performance was investigated by various trials; and results are reported in this work. Hence, it is recommended to use this test rig with suitable NFs in healthcare applications to predict the disease through breath analysis.

Many researchers have synthesized and investigated the gas-sensing performance of NFs such as ZnO,^21−23^ SnO_2_,^24^ Co_3_O_4_,^25^ and V_2_O_5_.^26−28^ Among these, the ZnO NFs are the preferred material for gas sensing due to their n-type semiconducting nature and wide bandgap of 3.37 eV. There are several methods for synthesizing ZnO, which include precipitation,^29^ hydrothermal,^30^ solvothermal,^31^ electrospinning,^32^ and sol–gel.^33^ Among these, electrospinning is a useful technique for manufacturing NFs. This method is cheaper and easier to obtain a long and continuous fiber. Generally, the following techniques are adopted to improve the sensing performance of the NFs: (i) the surface area-to-volume ratio of NFs can be improved by modifying the morphology; (ii) the electrochemical properties of NFs can be altered by suitable doping.^34,35^ These two techniques have been used by researchers to design NFs that improve the surface interaction of gas molecules, resulting in an enhanced gas sensing performance.

The response of the MO gas sensor is mainly dependent on the temperature. The sensing mechanism in MO materials exposed to the gas corresponds to the change in the magnitude of electric charge carriers induced by oxidation or reduction reactions on the surface. It indicates that the sensing capability of these sensors depends significantly on the surface area and adsorbing activities at the surface of the sensing material. In the present work, we fabricated a test device to evaluate the performance of ZnO MO-based sensors.

## 2 Experimental Section

### 2.1 Synthesis

**Figure 1:**
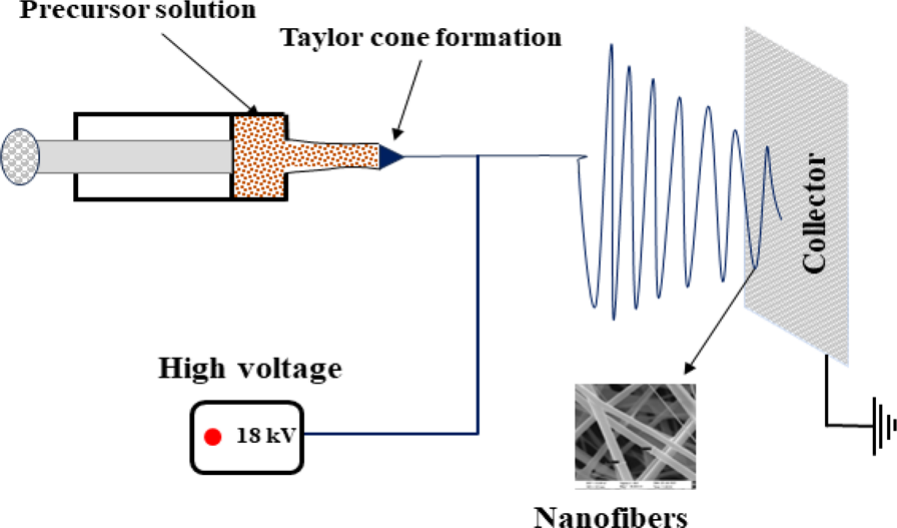
Schematic representation of the electrospinning process.

ZnO NFs were formed by pyrolyzing the precursor PVA/ZnAc_2_ ceramic fibers, and the sol–gel process was used to prepare a composite fiber. In this process, 7.5 g (15 w%) of polyvinyl alcohol (PVA) having molecular weight 115000 was magnetically stirred for 4 h at 100 °C. The 7.5 w% ZnAc_2_ was then added to the previously stirred PVA solution, and the resulting solution was magnetically stirred overnight at 50 °C. Further, the solution was loaded in a 10 mL syringe with its needle having 0.4 mm inner diameter and 20 mm length in an electrospinning setup, as shown in Figure 1. The syringe needle was connected to the positive terminal of a DC voltage (10–30 kV) and maintained a distance of 20 cm between the collector and the needle tip. Due to the high voltage, the solution that comes out of the syringe needle overcomes the surface tension, and the charged jet solution gets ejected from the needle, drawn as fiber, and deposited on the collector. The fibers thus obtained were dried at 60 °C under vacuum conditions for 3 h and pyrolyzed at 600 °C for 2 h.

### 2.2 Characterization

The crystalline structure of the NFs was investigated using powder X-ray diffraction (XRD, Rigaku MiniFlex 600, Japan) from 20 to 80° at a scanning speed of 0.5° m^–1^. The morphology of the NFs was studied using scanning electron microscopy (SEM, CARL, ZEISS, Germany). The optical spectra of the NFs were analyzed by an ultraviolet–visible spectrometer (UV–vis, Shimadzu-1800, Japan) at a range of 100–900 nm. The Brunauer–Emmett–Teller (BET) technique was adopted to measure the specific surface area of the NFs using a surface area analyzer (Smart Sorb 92, Smart Instruments Co. Pvt. Ltd., India). The Fourier transform infrared spectroscopy (FTIR, Shimadzu IRSpirit, Japan) spectra for as-spun and ZnO NFs were analyzed from 400–4000 cm^–1^ with a resolution of 1 cm^–1^.

### 2.3 Design, Fabrication of Gas Sensor Test Rig, and Measurement

**Figure 2:**
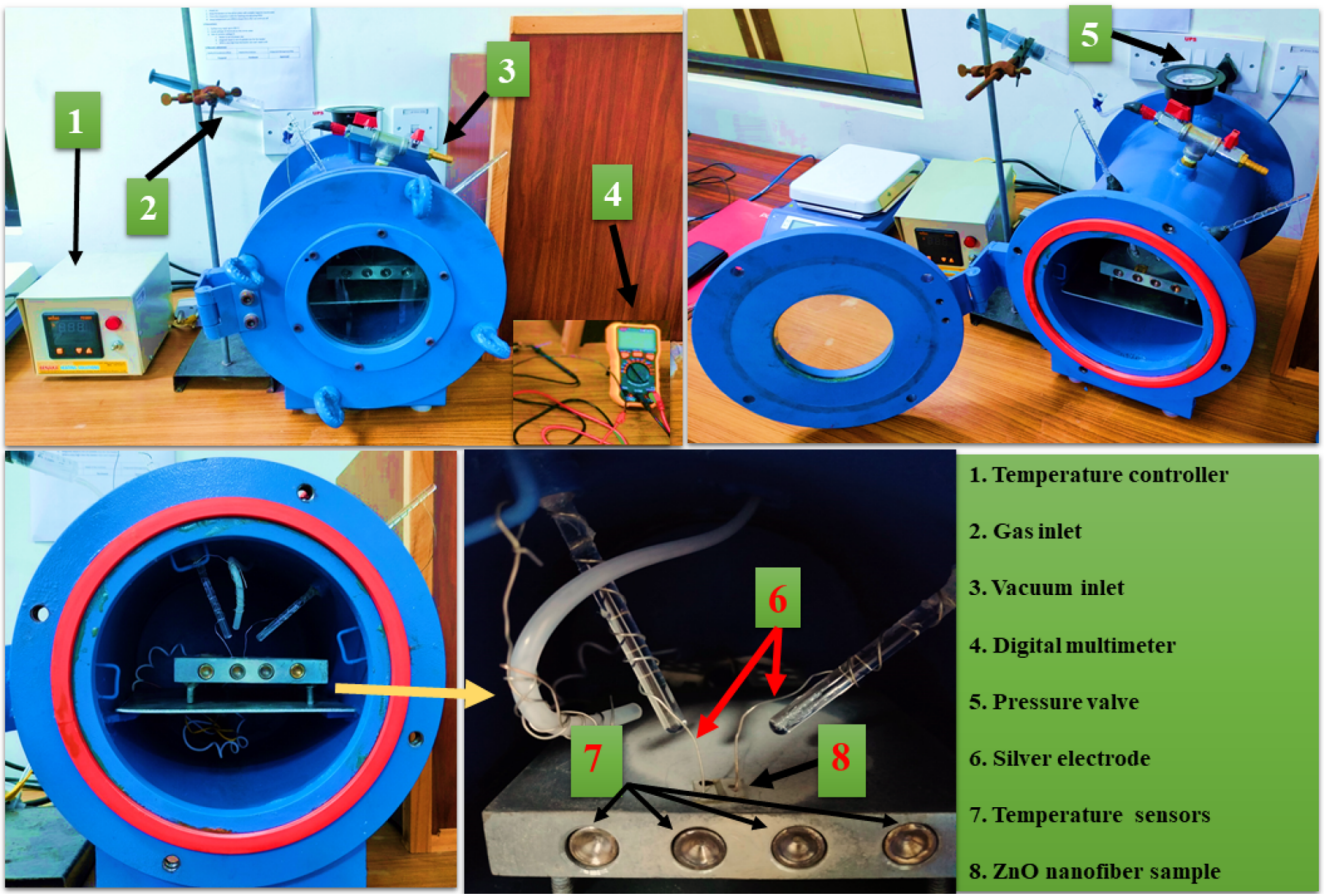
Gas sensing setup.

An indigenous test device was designed and fabricated for a gas volume capacity of 20 L with the facility to measure resistivity, as shown in Figure 2. The materials and their specifications used for the fabrication of the gas chamber are described in Table 2.

**Table 2:** Specification of the Gas Chamber

**materials used**	**specifications**
MS plate	300 mm diameter and 16 mm thickness
temp. control panel	temperature range (0–300 °C)
thermocouple	K-type diameter: 6 mm, length: 25 mm
silicon rubber blanket	12-in. diameter and thickness of 15 mm
vacuum gauge	0–600 mmHg
heater	high-density cartridge of diameter:12.5 mm, length: 125 mm, operating at 240 V and 300 W capacity

The ZnO/PVA fibers were spun on silica substrates and pyrolyzed at 600 °C for 2 h. After pyrolysis, the silver paste was coated along two edges of the ZnO NF mat and attached to two silver electrodes to measure the resistance. The target gases were injected with a microinjector into the closed chamber. The resistance of the ZnO NFs was measured as a function of the working temperature until a steady value was achieved using a digital multimeter. The measurements were carried out at various temperatures ranging from room temperature to 350 °C. The sensitivity (*S*) of the sensor is defined as the ratio of Ra/Rg, where Ra is the resistance in air and Rg is the resistance in the presence of the test gas. The chamber outlet was connected to an outlet pump to reduce the amount of leftover gas molecules.

## 3 Results and Discussion

### 3.1 Morphology Study

**Figure 3:**
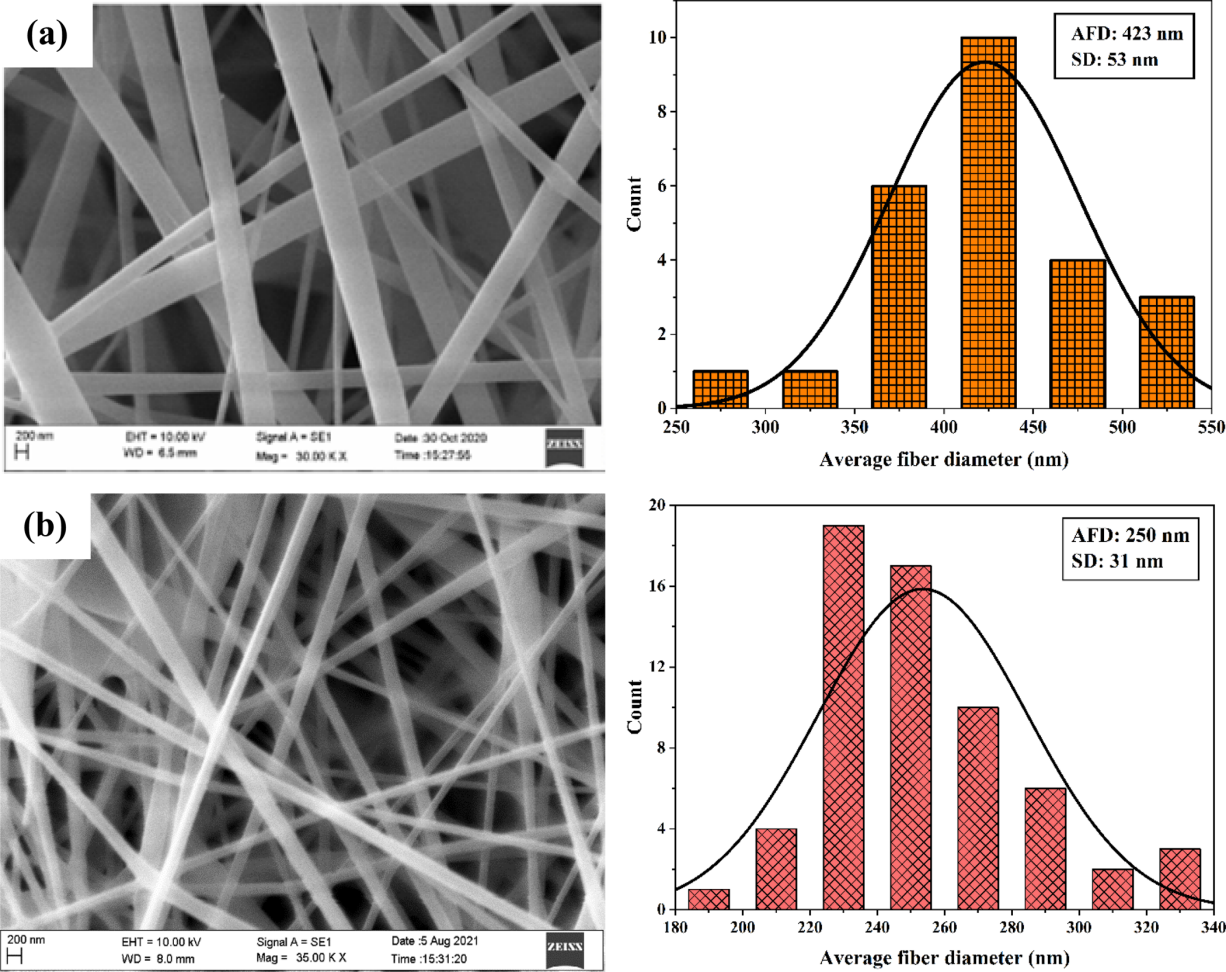
AFD of the NFs of (a) as spun and (b) pyrolyzed fibers.

**Figure 4:**
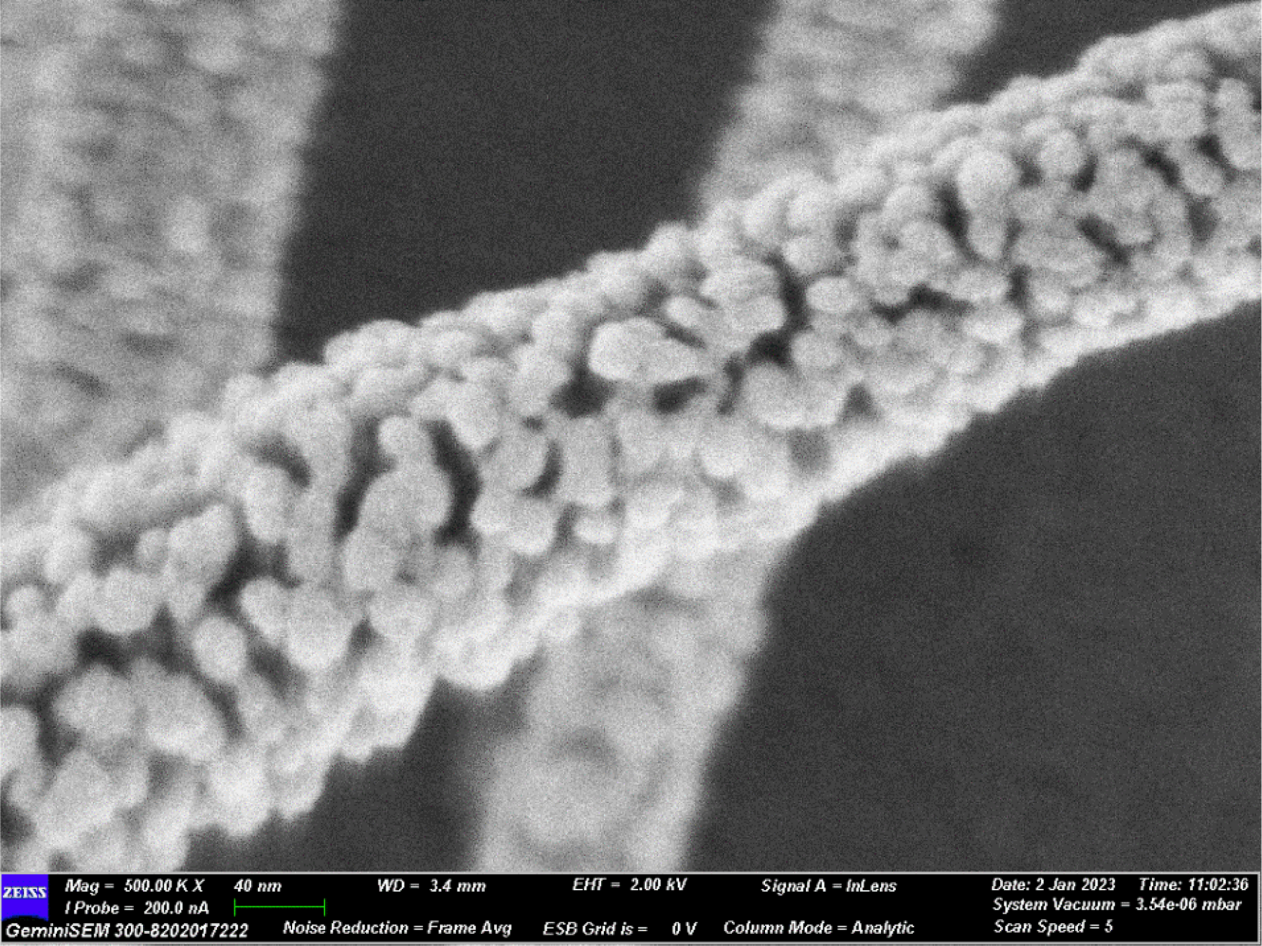
FESEM image of the ZnO NF.

Figure 3 depicts the SEM images of the as-spun and pyrolyzed NFs and their size distribution. A smooth and continuous nanofiber was observed for the as-spun NFs with an average fiber diameter (AFD) of 423 nm. The as-spun fibers were pyrolyzed at 600 °C and observed a rough surface with an AFD of 250 nm, as shown in Figure 3b. The PVA completely evaporated during the pyrolysis, leading to the shrinkage of the NFs. Figure 4 depicts the FESEM image of the ZnO NFs and shows the clear distribution and grain size of the NFs. From the literature, it was reported that Ghafari et al.^4^ observed a broader distribution of the NFs at 500 °C, with the reduction of NFs diameter from 232 nm before pyrolysis to 120 nm after pyrolysis. Similarly, Bose and Sanyal^36^ observed a uniform distribution of NFs with decreased fiber diameter from 550 to 380 nm at 500 °C. The images obtained by FESEM in the present work are in line with the results reported in the literature. However, the reduction size of the NFs depends on the concentration of the polymer and the pyrolysis temperature.

### 3.2 Structural Analysis

**Figure 5:**
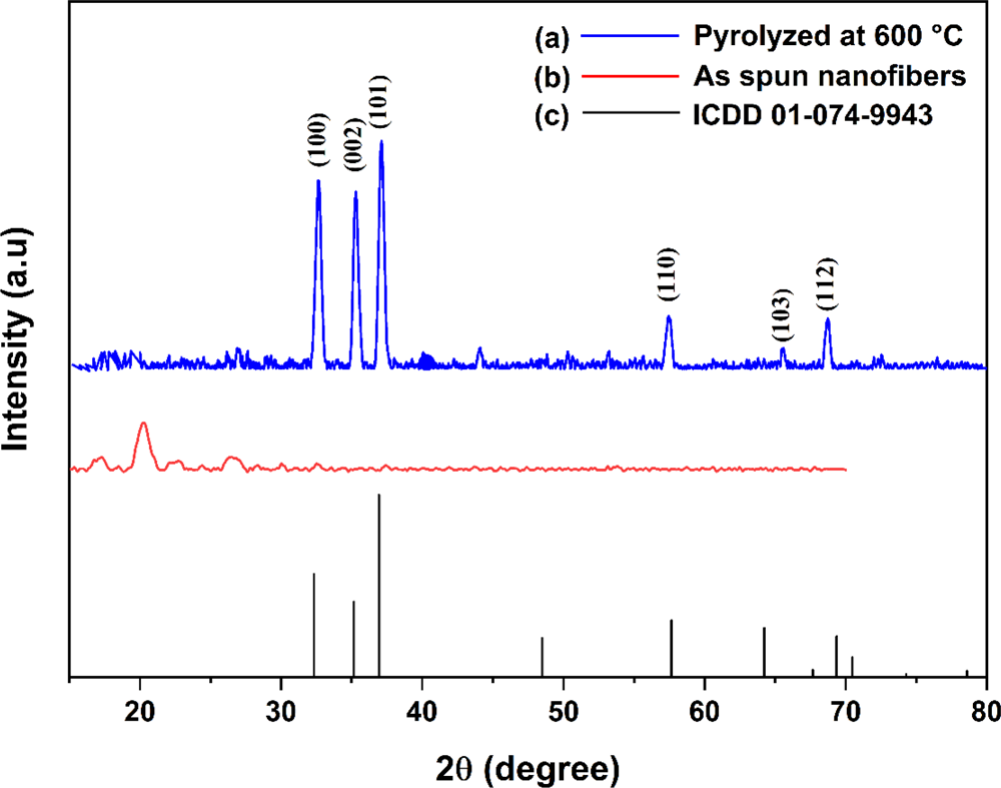
XRD patterns of the pyrolyzed NF.

The structural parameters of the as-spun NFs and ZnO NFs are determined using XRD analysis and reported in Figure 5. The NFs showed a polycrystalline nature with peaks corresponding to the hexagonal wurtzite structure of ZnO. The obtained values are compared with standard ICDD data (01–074–9943) of ZnO. The fibers showed (100), (002), (101), (110), (103), and (112) peaks with maximum intensity along the (101) plane corresponding to the *c*-axis orientation. The crystallinity of the fibers can be determined by using eq 1.
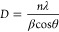
1

The crystallite size of the NFs is found to be 23 nm. The lattice parameters a and c are found to be 3.2 and 5.21 Å, matching with the standard ZnO values. Senthamizhan et al.^37^ also found the hexagonal wurtzite structure of ZnO with patterns (101), (002), (101), (102), and (110) having sharp peaks at 600 °C. Similarly, Bose and Sanyal^36^ observed the crystalline phase of ZnO with (100), (002), and (101) planes indexed at 31.30°, 34.06°, and 35. 80°. Further, Das and Srinivasan^38^ reported sharp peaks of ZnO with the (101) plane for the fibers pyrolyzed at 600 °C. The present investigation confirms that the crystalline structure of ZnO NFs is similar to the results obtained by the researchers.

The specific surface area of the NFs is one of the key factors in the gas-sensing performance of the MO NFs. The ZnO NFs specific surface area was calculated by taking 1 g of the sample. The sample was first regenerated by heating to 150 °C to evaporate the moisture and initially adsorbed gases. Further, regenerated NFs were kept in a tube containing a gaseous mixture (70% helium and 30% nitrogen) and immersed in liquid nitrogen (LN_2_). The regenerated sample is then exposed to nitrogen gas, which adsorbs and creates a single molecular layer of N_2_ on the surface of the NF surface. After the adsorption process, the sample tube is immersed in room-temperature water, resulting in the desorption of N_2_ molecules. The surface area was determined by using a single-point BET equation by measuring the volume of N_2_ adsorbed. It was found that a high surface area of 5.4 m^2^ g^–1^ and pore volume of 0.013 cm^3^ g^–1^ were found for the ZnO NFs. This is attributed to the complete evaporation of PVA upon pyrolysis, and it is good evidence that ZnO NFs are most preferred for gas sensing.^39^

### 3.3 UV Spectroscopy

**Figure 6:**
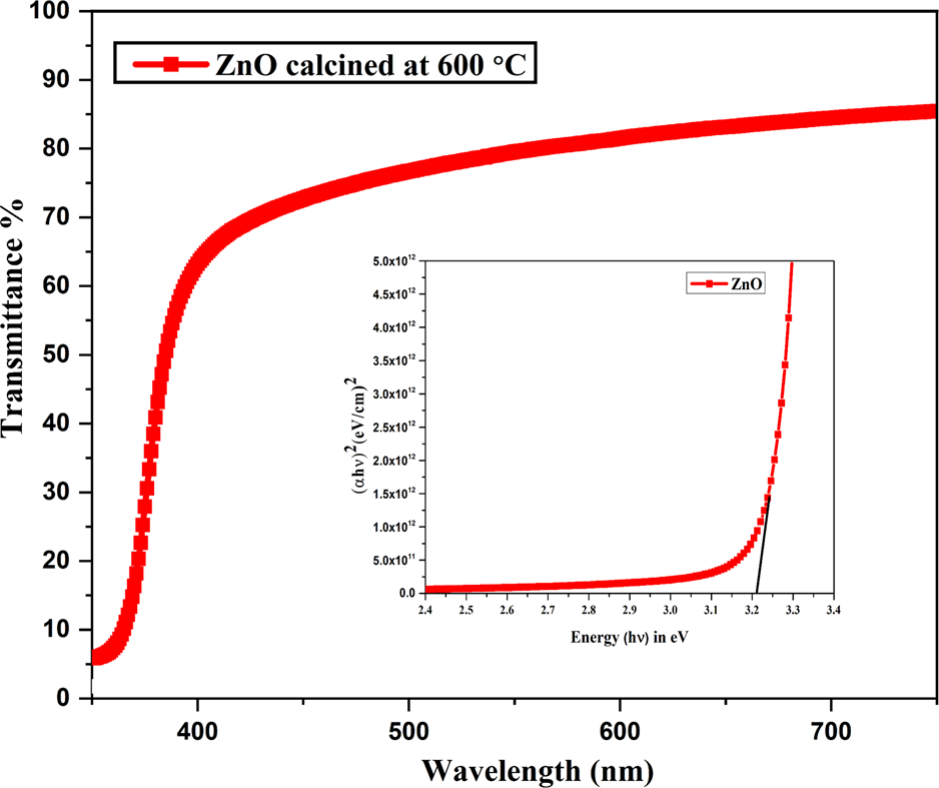
Transmission and band gap energy spectra of ZnO fibers.

The optical properties of the NFs are calculated using the UV–vis double-beam spectrometer. Figure 6 shows the transmittance spectra and bandgap of ZnO NFs pyrolyzed at 600 °C. The transmittance of the NFs was found to be 80% in the visible region. The absorption edge of the NFs was found to be 385 nm. The sharp absorption edge corresponds to better optical and structural properties of the fibers. The better transparency of the NFs is due to the increased crystallinity and lesser defects. This is also confirmed by XRD analysis. The lattice orientation of the NFs increased with increased crystallinity and decreased scattering of the light. The band gap of the NFs is calculated using eq 2.
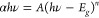
2where α is the adsorption coefficient, hν is the incident photon energy, *E_g_* is the optical band gap, and *n* is an index. The energy band gap of the NFs was found to be 3.22 eV, which matches with the theoretical value of ZnO. The variation in the band gap is due to the shift in the Fermi level because of the variation in the deposition parameters. The band gap of the fibers also depends on the crystallinity of the fibers, and it decreases as the crystallinity of the NFs increases.

### 3.4 FTIR Spectroscopy

**Figure 7:**
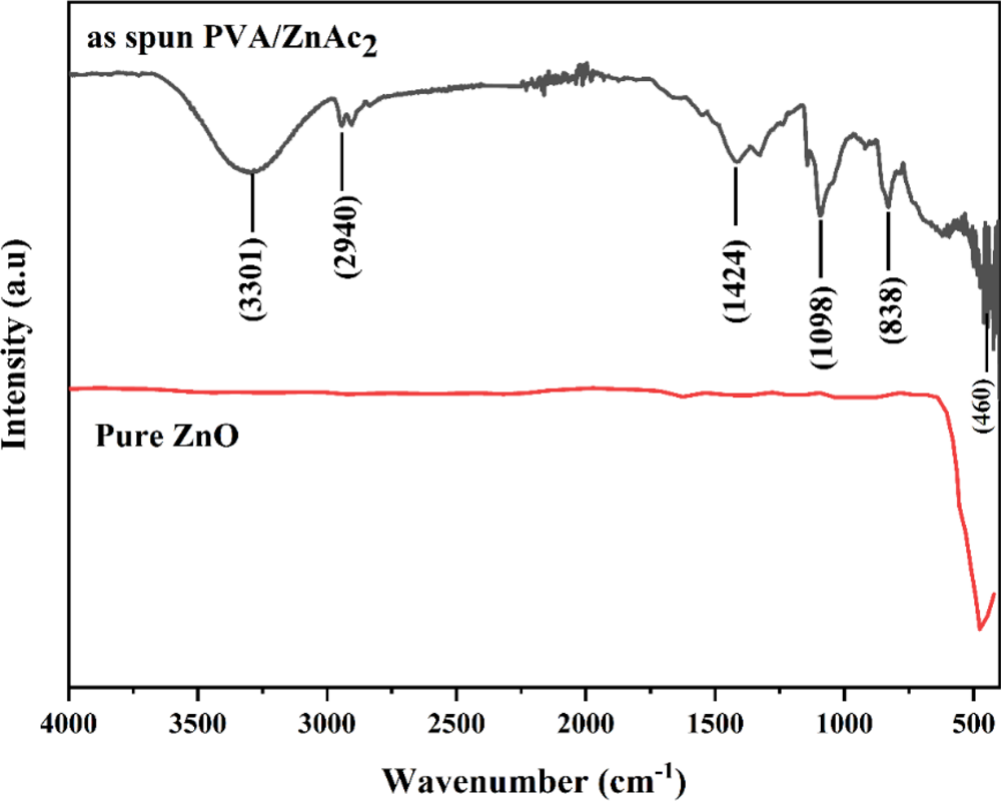
FTIR spectra of spun PVA/ZnAc_2_ and ZnO NF.

The FTIR analysis was performed for as-spun PVA-ZnAc_2_ NFs to determine the interaction between ZnAc_2_ and PVA and the chemical composition of ZnO NFs. Figure 7 shows the spectra of the as-spun PVA-ZnAc_2_ NFs and ZnO NFs. The as-spun NFs spectra show two peaks at 2800–3500 cm^–1^ intervals. The peak at 3301 cm^–1^ is attributed to the O–H bond, and the band at 2940 cm^–1^ corresponds to the C–H bonds.^40^ Similarly, the peaks at 1424, 1098, and 838 cm^–1^ are assigned to the C–C and C–O groups of PVA.^41^ After the as-spun NFs were pyrolyzed, all the peaks associated with the organic and the water molecules disappeared, indicating the complete decomposition of the organic residual from the as-spun NFs. The spectra for pure ZnO NFs show a vibration band at around 476 cm^–1^ corresponding to the ZnO samples.^42^

### 3.5 Gas Sensing Studies

#### 3.5.1 Gas Sensing Mechanism

The sensing mechanism of an n-type MO semiconductor is explained by the space-charger layer method,^43−45^ which is related to the change in sensor resistance when the sample is exposed to air and gas atmospheres. When the ZnO NFs surface is exposed to air, some of the oxygen molecules are chemisorbed, forming chemisorbed oxygen species (O_2_^–^, O^–^, and O^2–^). Due to this, electrons get extracted from the conduction band of the material under different operating temperatures, and the following reactions take place.^46^
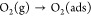
3
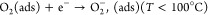
4
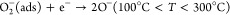
5
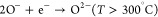
6

Similarly, when the ZnO NFs are in contact with reducing gases such as H_2_, CO, and CH_4_, it reacts with the chemisorbed oxygen molecules, decreasing the number of oxygen molecules. This led to a decrease in the electron extraction from the conduction band, thereby reducing sensor resistance. Similarly, when the ZnO NFs are exposed to oxidizing gases such as NO_2_, O_2_, O_3_, and H_2_SO_4_, the chemisorbed oxygen molecules are oxidized, increasing the number of oxygen molecules. This increases the number of electrons extracted from the conduction band, thereby increasing the sensor resistance.

#### 3.5.2 Gas Sensing Performance of NFs

**Figure 8:**
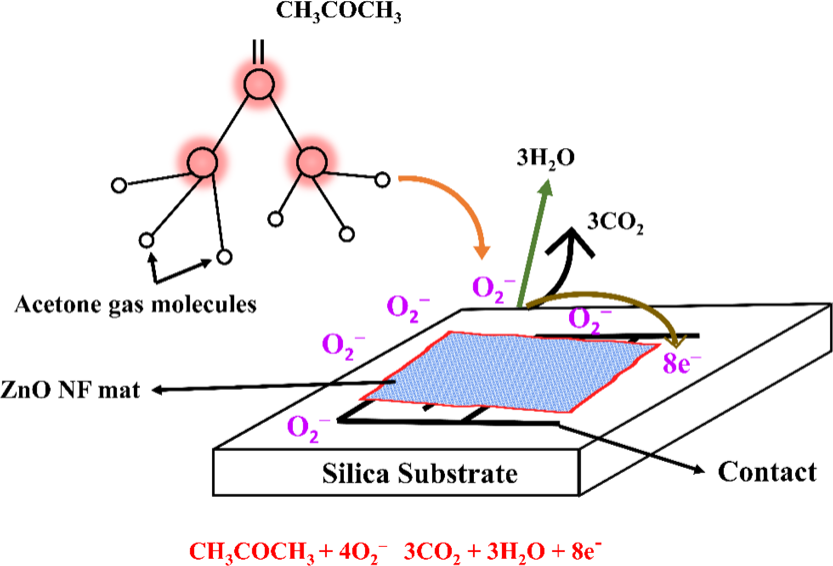
Sensing mechanism for acetone vapor.

When the ZnO NFs are exposed to acetone, the gas accumulates on the surface of ZnO, and a reducing reaction occurs between acetone gas molecules and the chemisorbed oxygen ions. Figure 8 depicts the reaction of acetone molecules upon exposure to the surface of ZnO NFs. This is explained by the following reaction.
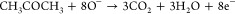
7

**Figure 9:**
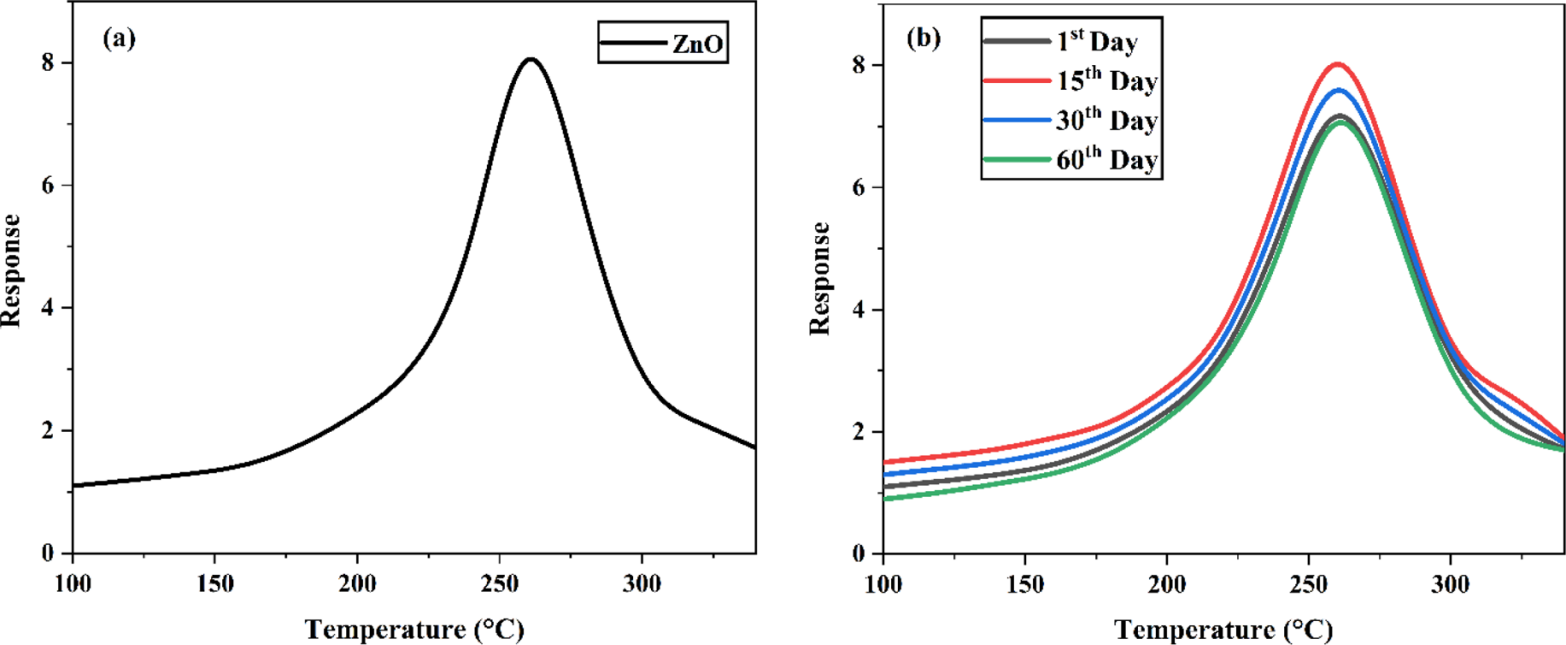
(a) Response and (b) stability of ZnO NFs to acetone vapor.

During this process, the trapped electrons at the interface between the chemisorbed oxygen ions and the ZnO surface are released back to the conduction band of the sensing material. This diminishes the electron depletion region at the interface and the potential barrier decreases, resulting in a significant decrease in resistance. Figure 9a displays the sensing response of the ZnO NFs to acetone vapor with respect to the working temperature.

The response increased with the operating temperature to 260 °C and decreased with an increase in the operating temperature. This phenomenon can be explained by the kinetics and mechanics of the adsorption and desorption of gas on the ZnO surface.^47^ When the operating temperature is lower, the oxygen molecules will be in the physisorption state, where the adsorption attraction is weaker, leading to low response. At higher operating temperatures, some of the adsorbed gas molecules escape before the reducing reaction due to their enhanced activation, resulting in a higher response. The ZnO NFs showed the highest response at a temperature of 260 °C. The response and recovery time of the ZnO NF sample were about 120 and 80 s for the 50 ppm acetone.

Further, the ZnO NFs were investigated by exposing them to acetone vapor after various time intervals such as the 1st day, 15th day, 30th day, and 60th day at room temperature to 350 °C to study the stability. Figure 9b shows the response of the ZnO NFs at different intervals and found that the proposed ZnO NFs have long-term stability.

To cross-check the results obtained by the proposed test rig, we identified similar research work carried out by the various researchers and listed in Table 3 along with the present work. It was found that the results obtained from the proposed test rig are in line with the results listed in the literature, even at low concentrations of gas showed higher response at lower temperature.^48^ The operating temperature is also close to those of the literature valves. However, a few cases observed variations in response and operating temperature for doped ZnO NFs.

**Table 3:** Pure ZnO and Doped ZnO NF Responses to Acetone Vapor

		gas sensing parameters				
material combination		type of gas	response (Ra/Rg)	con. (ppm)	temp (°C)	ref
ZnO	pure	acetone	2.3	200	400	(48)
	reduced graphene oxide		4		200	(48)
ZnO	manganese	acetone	89	450	340	(49)
ZnO	cerium	acetone	71	500	230	(50)
			75	100	260	(51)
ZnO	indium	acetone	19.3	50	200	(52)
ZnO		acetone	81	100	325	(53)
ZnO	lanthanum	acetone	48	100	340	(54)
ZnO	cobalt	acetone	56	500	360	(55)
ZnO		acetone	8	50	260	present work

**Figure 10:**
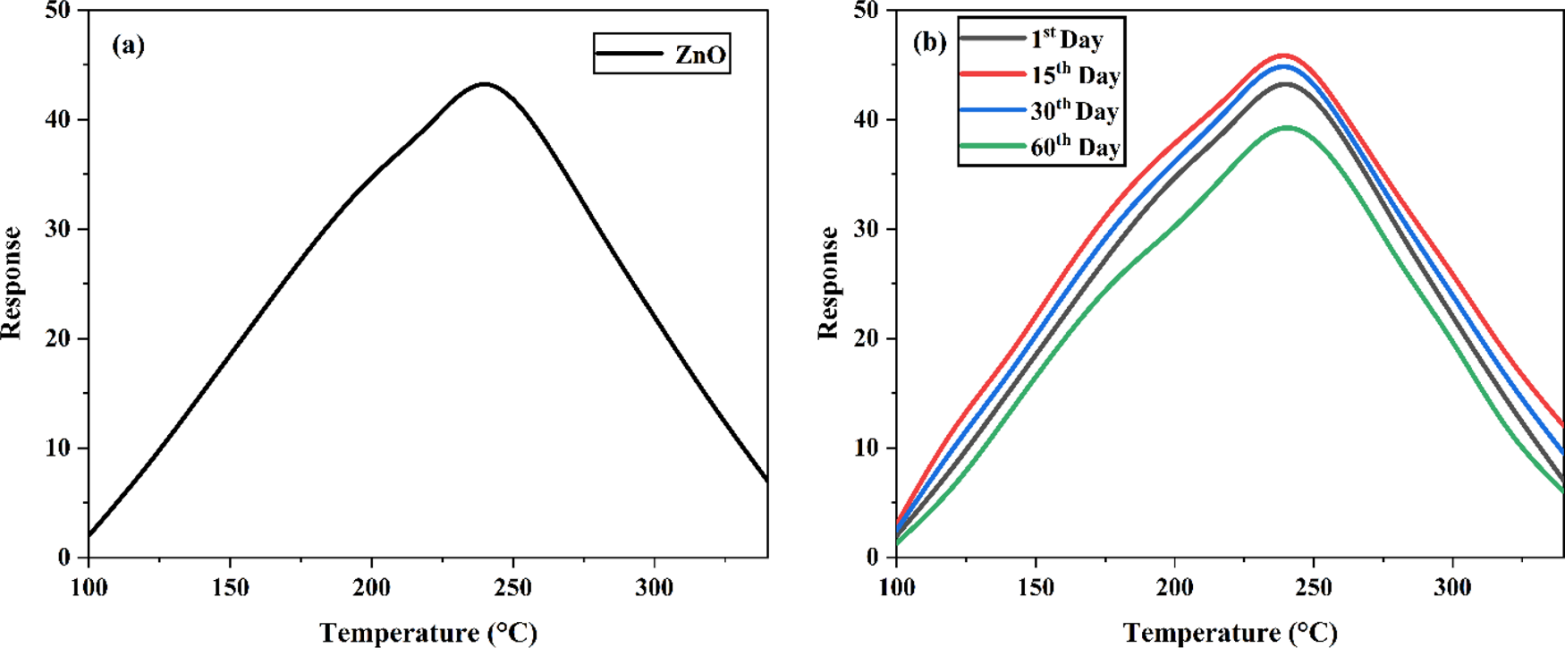
(a) Response and (b) stability of ZnO NFs to ethanol vapor.

The sensing mechanism of ethanol is similar to that of acetone. The responses of ZnO NFs were measured by exposing the surface of NFs to 50 ppm of ethanol under various temperatures. Figure 10a shows an increased response with the temperature and a maximum response at 240 °C; further increase in temperature decreased the response. This is because, at temperatures up to 240 °C, the absorbed ethanol molecules do not have sufficient energy to overcome the activation energy barrier, thereby failing to react with the absorbed oxygen species. Further, as the temperature increases, some absorbed oxygen species escape before the reaction, decreasing the response. The increase and decrease in gas response with the operating temperature are also attributed to the long NF structure, which has a high surface area, increasing the adsorption of oxygen species and promoting the reaction on the fiber surface at a lower temperature. The ZnO NFs showed the highest response value of 31.74 at 240 °C. Table 4 denotes the response of ZnO NFs for ethanol gas, which was studied by various researchers. The higher response is attributed to the larger specific surface area, which will absorb more oxygen molecules to react with the ethanol molecules. The following reaction took place on the ZnO surface:
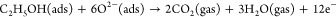
8

**Table 4:** Pure ZnO and Doped ZnO NF Responses of Ethanol Vapor

		vapor sensing parameters			
material combination		response (Ra/Rg)	con. (ppm)	temp (°C)	ref
ZnO	Er	37.3	20	240	(56)
ZnO	Al	8.6	100	250	(57)
ZnO	SnO_2_	423	4000	300	(58)
ZnO	SnO_2_	83	20	260	(59)
ZnO	SnO_2_	392.29	100	200	(60)
ZnO	TiO_2_	50.6	500	320	(61)
ZnO	TiO_2_	15.7	100	280	(62)
ZnO		31.74	50	240	present work

The response and recovery time of the ZnO NFs were about 40 and 30 s to 50 ppm ethanol vapor. The stability of the sensor was investigated for the 1st day, 15th day, 30th day, and 60th day time intervals. After four cycle response measurements to 50 ppm ethanol at 240 °C, the response of the ZnO NFs has no significant fluctuation, indicating good stability (Figure 10b).

**Figure 11:**
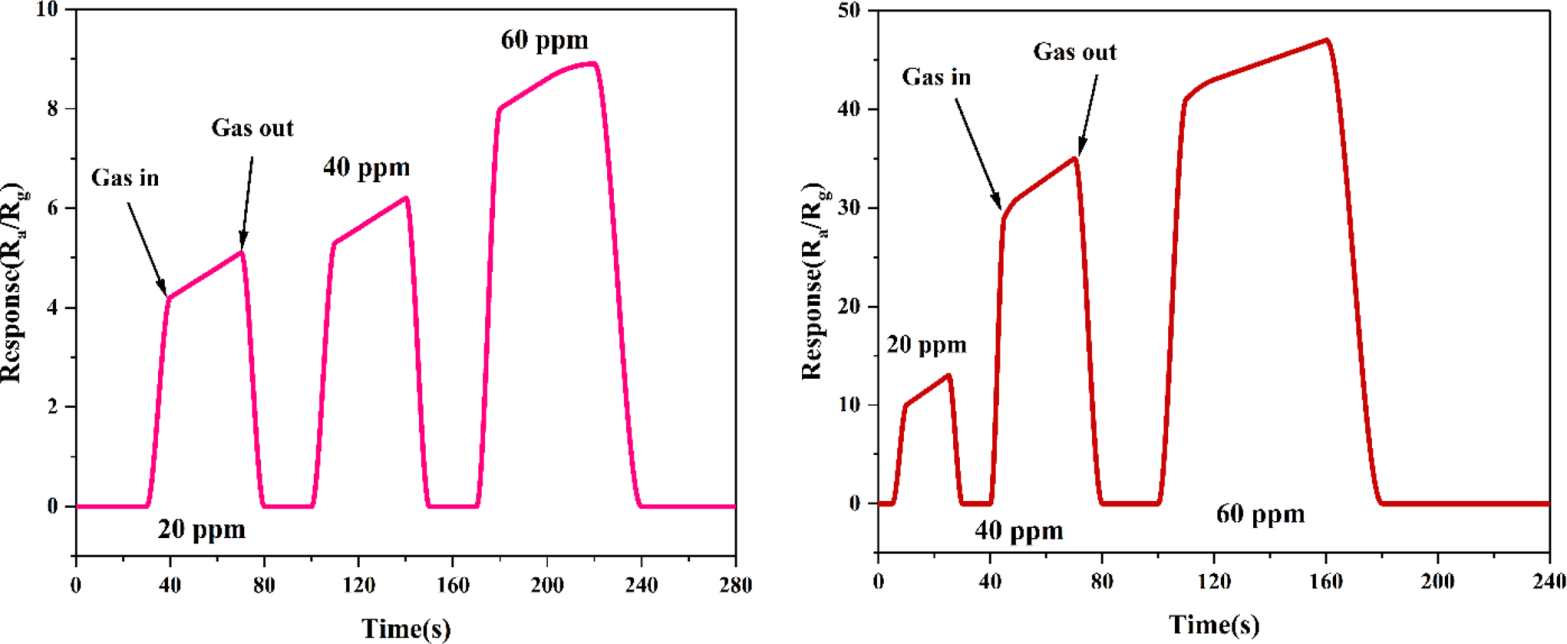
Transient responses of ZnO NFs for various concentrations of acetone and ethanol vapors.

Figure 11 shows the transient responses of ZnO NFs in acetone and ethanol vapors at different concentrations. It can be found that the response increases with the increase in the concentration of the gas with respect to time.

**Figure 12:**
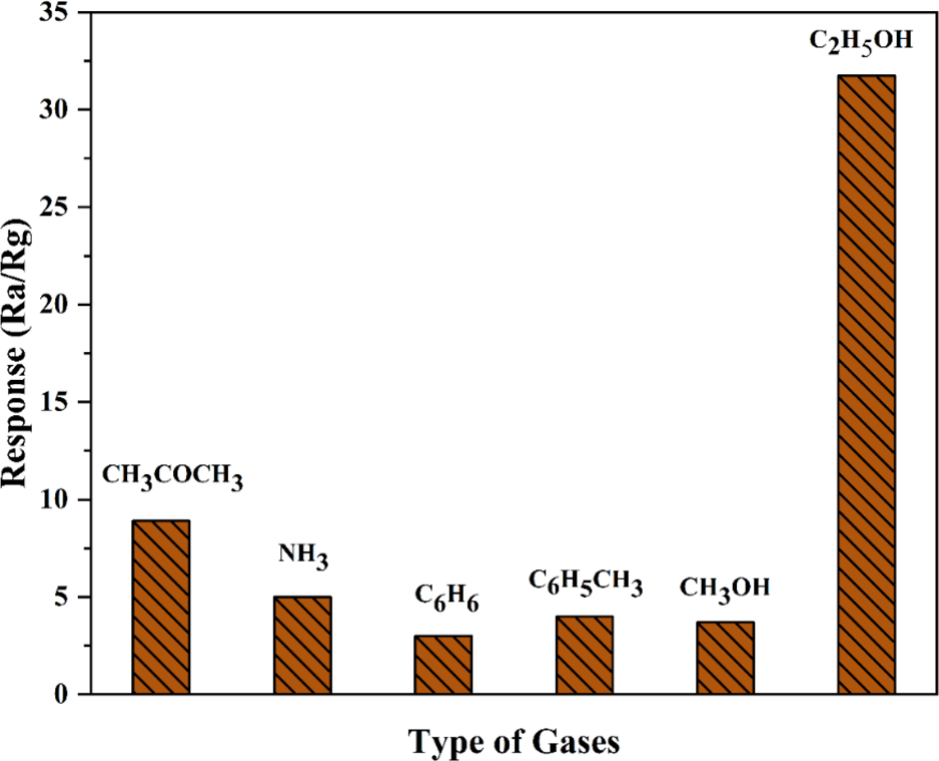
Sensitivity of the ZnO nanostructured sensors to a variety of VOCs.

Cross-sensitivity is another fundamental characteristic of gas sensors. The ZnO sensor was exposed to 50 ppm of different gases (CH_3_COCH_3_, C_2_H_5_OH, NH_3_, C_6_H_6_, and CH_3_OH). Figure 12 shows the selectivity of the ZnO sensors for several gases at 240 °C with a vapor concentration of 50 ppm. All the ZnO sensors show a higher response to ethanol vapor compared to other vapors. The responses to C_2_H_5_OH, CH_3_OH, C_6_H_6,_ CH_3_COCH_3_, and NH_3_ are 31.74, 3.7, 3.12, 8.9, and 5 respectively.

## 4 Conclusions

It is concluded from the results that PVA completely evaporated from the electrospun PVA/ZnAc_2_ NFs and formed pure ZnO NFs pyrolyzing at 600 °C. The AFD of the NF decreased after pyrolysis. The XRD results confirmed the ZnO NFs exhibited a wurtzite structure with their preferred orientation of the (101) plane. The energy band gap was found to be 3.22 eV. The gas sensing response of ZnO NFs obtained from the test rig is in line with the reported values from the researchers. A high response was found for acetone and ethanol vapor at operating temperatures of 260 and 240 °C, respectively. The response for ethanol was better as compared to acetone at 260 °C. The proposed gas sensing test rig can be used for testing the ZnO NF performance for designing gas sensors. Also, the test rig can be improved and may be used to diagnose diseases by investigating the concentration of various gases in the human breath. However, few clinical trials are required to standardize to use in the healthcare sector.

## Data Availability

All the data that support the findings of this study are available within the article.
